# Flourishing of Rural Adolescents in China: A Moderated Mediation Model of Social Capital and Intrinsic Motivation

**DOI:** 10.3390/ijerph18158158

**Published:** 2021-08-01

**Authors:** Sijia Guo, Diyang Qu

**Affiliations:** 1 College of Public Administration and Humanities, Dalian Maritime University, Dalian 116000, China; sijiaguo@dlmu.edu.cn; 2 Department of Social and Behavioural Sciences, City University of Hong Kong, Hong Kong, China

**Keywords:** adolescents, rural areas, flourishing, social capital, intrinsic motivation

## Abstract

Flourishing, an indicator which reflects one’s emotional status and ability to function, is an important antecedent for adolescents’ later psychological and physical development. This study aimed to shift the research focus on rural adolescents from a deficit-based orientation to a strength-based orientation by integrating the effects of individual and social factors. Altogether, 995 Chinese rural adolescents (569 boys and 426 girls, Mage = 12.56 years) completed a self-report questionnaire which included the Ego Identity Scale, Intrinsic Motivation Scale, Social Capital Scale and Flourishing Scale. The results indicate that: (1) ego identity exploration has a positive effect on rural adolescents’ flourishing; (2) intrinsic motivation has an indirect effect on the relationship between ego identity exploration and flourishing; and (3) social capital may moderate the indirect effect by attenuating the relationship between ego identity exploration and intrinsic motivation. The findings highlight the importance of constructing a more comprehensive framework by integrating individual and social perspectives to understand and foster the flourishing of rural adolescents. Meanwhile, it is necessary to incorporate the strengths of family, school and social organizations in order to enhance rural adolescents’ flourishing.

## 1. Introduction

Adolescence is the transitional stage between childhood and adulthood. Children entering adolescence go through many challenges that may directly influence their mental health; for example, some may experience a higher level of depressive symptoms or anxiety [1]. Understanding the factors that promote their positive adjustment amid this process is an urgent public health issue. In recent decades, due to rapid urbanization, the gaps between urban areas and rural areas in China have gradually been enlarged. Given the limited resources in rural areas, rural adolescents may live with fewer social services, barriers to healthcare access and worse educational facilities than those in urban areas [2,3]. As such, numerous empirical studies have paid attention to Chinese rural adolescents’ maladjustment outcomes. For instance, Bilige et al. [4] explored the severe school dropout phenomenon among rural adolescents and found that those vulnerable dropout adolescents had a relatively low level of interpersonal relationships and poor school adjustment. Other studies have also pointed out that rural adolescents had poorer mental health and exhibited more delinquent behaviors than urban adolescents [5,6]. 

The traditional deficit-based approach that primarily focused on the negative consequences and risk process of adversity among vulnerable adolescents has been criticized for its ineffectiveness [7]. In recent years, a growing number of studies have shifted the focus from the problematic outcomes to the individual’s growth in terms of personal strength, potential and competence [8,9]. In other words, the strength-based approach upholds humanism, which highlights the individual’s plasticity and potential. Accordingly, the academic field has gradually shifted its attention to the positive adjustment outcomes of adolescents [10]. In light of the strength-based approach, this study aims to focus on rural adolescents’ flourishing. 

Flourishing goes beyond the confines of simple well-being; it is a state in which all aspects of one’s experience are good [11,12]. It is an integrative concept including multiple dimensions, namely, hedonism, psychological functioning and social functioning [13]. Hedonism refers to one’s life satisfaction and degree of happiness, which are the fundamental components of well-being. Psychological functioning indicates one’s inner growth by fulfilling one’s potential [14]. Moreover, social functioning indicates one’s social well-being, which is composed of social integration, social acceptance, social contribution, social actualization and social coherence [13]. Flourishing is an important determinant for adolescents’ development, and a large body of literature has explored the effects of flourishing. For instance, Keyes [15] measured the mental status of American youth ranging from 12 to 18 years old. The findings indicated that the adolescents with a higher level of flourishing functioned better than other adolescents. Similarly, Witten et al. [16] pointed out the importance of flourishing and its long-term effects on adolescents’ cognitive, emotional and social development. As the above examples illustrate, empirical studies have claimed that flourishing could exert positive and strong effects on adolescents’ development and psychosocial growth. From this perspective, promoting adolescents’ flourishing to pursue their positive development is necessary. In other words, a better understanding of the determinants contributing to these “better-than-expected” outcomes may inform the direction of intervention development. 

One’s flourishing is intertwined with diverse protective or detrimental factors, but studies focusing on these factors are inadequate [11,17]. When considering adolescents who are experiencing the transition from childhood to adulthood by separating from their parents psychologically, lifespan theory indicates that ego identity exploration is essential for generating adolescents’ positive adjustment outcomes, including flourishing. Ego identity exploration, a key developmental task, refers to the extent to which adolescents develop a clear and unique view of themselves and of their identity [18]. There are two means of ego identity exploration: reconsideration of commitments and in-depth exploration [19]. Specifically, reconsideration of commitments is when adolescents explore their identities by comparing current commitments with other alternatives and then deciding which one to choose. In-depth exploration is the process of continuously monitoring current commitments. No matter which means the individual experiences, ego identity exploration is a necessary stage that aids young people in discovering their strengths, weaknesses, uniqueness, potential and interests. A balanced and coherent ego identity exploration may be conducive to adolescents’ psychological functioning [19]. Nevertheless, studies focusing on the relationship between ego identity exploration and adolescents’ flourishing are still insufficient, and their results are ambiguous [20,21,22]. Therefore, during adolescence, the association between ego identity exploration and adolescents’ flourishing remains to be investigated and unveiled. 

### 1.1. Intrinsic Motivation as a Mediator

Intrinsic motivation stands for one’s spontaneous conscious endeavor to unearth the world to achieve self-actualization, which is viewed as an essential antecedent for adolescents’ psychological development outcomes [23]. Humanistic theories view the individual as a unit with unique strengths and potential. These theories postulate that it is natural for human beings to pursue a flourishing life. In line with this, self-determination theory highlights that intrinsic motivation involves carrying out a behavior because the activity itself is interesting and spontaneously satisfying [24], which is important for one’s functioning and positive emotional state across different cultural contexts, lifespans and life domains [25]. A growing number of empirical studies have examined the effects of one’s intrinsic motivation on his or her well-being [26]. Interestingly, these empirical studies found that intrinsic motivation is, indeed, a notable protective factor promoting one’s well-being and development [27,28]. For instance, Taylor et al. [29] pointed out that intrinsic motivation showed a significantly positive effect on students’ academic performance and achievement. However, most current studies focus on the individual in the Western cultural context, which values individualism. This generates the question as to whether intrinsic motivation displays a similar effect on people from other cultures, such as those from a collectivistic cultural context. Given China’s traditional Confucian culture, Chinese people may internalize their cultural norms (e.g., shě jǐ wèi rén, make personal sacrifices for the public benefit) which then may guide their behaviors in daily life [30]. From this perspective, how intrinsic motivation influences the flourishing of people from different cultural backgrounds needs to be further investigated. 

The nature of intrinsic motivation may be concordant with its ego identity exploration. Accordingly, Kaplan & Flum [31] argued that it is necessary to integrate identity theory when examining adolescents’ intrinsic motivation, as the existing empirical studies which focused on intrinsic motivation overlooked the importance of identity status. In line with the ego identity exploration theories, if the individual experiences a well-balanced exploration process, they will become intrinsically motivated. For instance, Deci & Ryan [32] mentioned that children would play with what they are interested in during their ego identity exploration, which is driven by intrinsically motivated behavior. Therefore, during the process of ego identity exploration, adolescents’ intrinsic motivation would be influenced to some extent. 

Regarding the mechanism among ego identity exploration, intrinsic motivation and flourishing, intrinsic motivation may possibly produce the indirect effect between ego identity exploration and flourishing. Specifically, during the process of ego identity exploration, adolescents may search for their own identities by comparing dimensional alternatives and then recognizing their own characteristics, interests, potential and uniqueness, which facilitates intrinsic motivation. In other words, when adolescents gradually find out what they are interested in and good at, they may have the courage and passion to behave by their own volition and achieve their own goals [33]. Furthermore, the individual may experience a positive emotional state and functioning when he or she is in an elevated state of intrinsic motivation. Herein, there may be an indirect effect of ego identity exploration on flourishing via intrinsic motivation. 

### 1.2. Social Capital as a Moderator

The individual is not isolated but is embodied within a social context. Self-determination theory highlights the role of the social context in promoting adolescents’ intrinsic motivation [34]. Adolescents are largely influenced by their access to social resources and relations, including their parents, peers and teachers [35]. Specifically, psychodynamic theories emphasize that adolescents consider both what they want to be and what they should be (this is largely influenced by their surroundings, including family requirements, expectations from others and social norms) when they explore their identities [36]. The former reflects the true self, while the latter represents the influence of the societal context [18]. In particular, Chinese adolescents are influenced by aspects of traditional Chinese culture, such as filial piety—a central Confucian virtue in social ethics which addresses the attitudes of obedience, devotion and care towards others. 

Social capital may also play an important role in this indirect effect by moderating the association between ego identity exploration and intrinsic motivation. Social capital, referring to one’s social networks and resources, could be effective in fulfilling one’s needs and desires [37]. For example, the overwhelming support from Chinese rural adolescents’ social networks may exert too much influence on adolescents and therefore limit the relationship between their ego identity exploration and intrinsic motivation. On the other hand, appropriate social capital may provide more opportunities or space for adolescents to realize their potential selves, which would strengthen the effect of ego identity exploration on intrinsic motivation [38]. For instance, [39] found that relatively acceptable doses of mobile social media use provided a new platform for adolescents embedded within more opportunities to explore and rethink their own identities, which further influences adolescents’ academic learning motivation. Although few studies have addressed the associations between social capital and intrinsic motivation, a large research gap lies among these three variables, which needs to be addressed. 

### 1.3. The Present Study 

Flourishing is vital for adolescents’ development and is attracting tremendous attention from various disciplines, including positive psychology, family studies and public policy. For Chinese adolescents, especially rural adolescents, studies focusing on their flourishing status are not abundant, and most previous studies only paid attention to the unidimensional association rather than considering multidimensional intertwined linkages. 

Taking the characteristics of adolescents into account, adolescents’ flourishing is influenced by different layers, including the individual’s characteristics and social context. As mentioned above, adolescence is a transition period during which adolescents are in a fluctuant state, searching for their own identities. Therefore, as an inevitable process, ego identity exploration is an important factor in adolescents’ flourishing. Additionally, as an inborn feature, every adolescent is motivated to pursue a flourishing life by exploring and accomplishing one’s interests and goals. In other words, intrinsic motivation naturally contributes to adolescents’ flourishing. In view of the nature of ego identity exploration and intrinsic motivation, it is plausible that the degree of one’s identity exploration may have an impact on one’s intrinsic motivation and thereby influence personal flourishing. In other words, this is the portion of the relationship between ego identity exploration and flourishing that may go through intrinsic motivation. Furthermore, according to self-determination theory, the individual’s intrinsic motivation is affected by the social context as well. Social capital, as a significant indicator reflecting the condition of the social context, influences one’s intrinsic motivation.

**Figure 1:**
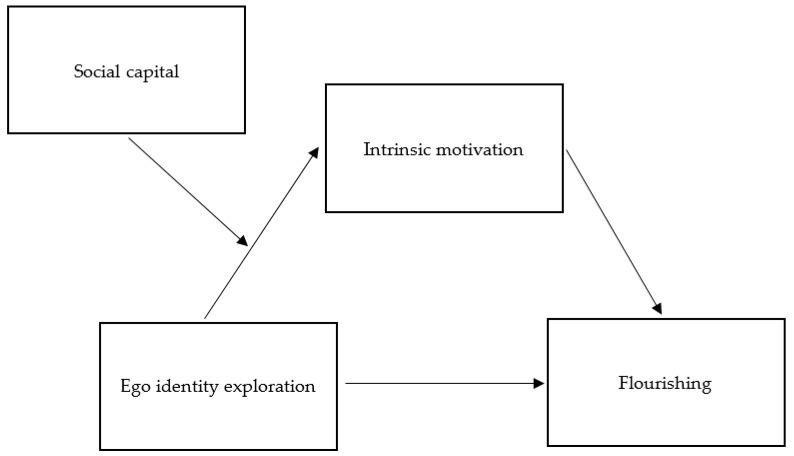
A hypothesized moderated mediation model.

To this end, we propose the following hypotheses (See Figure 1):

**Hypotheses** **1 (H1).** *Ego identity exploration contributes to rural adolescents’ flourishing.*

**Hypotheses** **2 (H2).** *Intrinsic motivation mediates the relationship between ego identity exploration and flourishing among rural adolescents.*

**Hypotheses** **3 (H3).** *Social capital modifies the effect of ego identity on intrinsic motivation among rural adolescents.*

## 2. Method

### 2.1. Participants

A total of 1022 participants were recruited from five primary schools and three junior high schools in five counties of Liaoning, China. After removing 27 invalid samples, a total of 995 questionnaires were obtained, with a response rate of 97%. The age of participants ranged from 7 to 19 (M = 12.56, SD = 1.38); a total of 57% (N = 569) were boys. Data collection occurred at the end of 2017. Permission was obtained from the schools. Written informed consent was obtained from all participants and their parents or guardians. Anonymity, confidentiality and the study’s voluntary nature were guaranteed. Participants later completed a self-administered questionnaire with the assistance of school teachers. The [BLINDED FOR REVIEW] approved the study. 

### 2.2. Measurement

Demographic Details. Participants answered questions about their demographic backgrounds, including their sex (i.e., 0 = boys, 1 = girls), age and family income (i.e., 0 = below CNY 100,000, 1 = CNY 100,000–120,000, 3 = above CNY 120,000 per year). 

Ego Identity Exploration. Ego identity exploration was measured using the 12-item Ego Identity Process Exploration instrument (EIPE, [40]). Example items are, “*My values are likely to change in the future*” and “*I have re-examined many different values in order to find the ones that are best for me*”. Items were rated on a 5-point Likert scale. A higher score indicated a higher level of ego identification. In the current study, this measure showed acceptable internal reliability, with a Cronbach’s alpha of 0.69. 

Intrinsic Motivation. Intrinsic motivation was measured using the Intrinsic Motivation Scale [41]. An example item is “*In the past two weeks, in daily life, I have liked engaging in hard activities because it is a challenge*”. Items were rated on a five-point Likert scale. A high score indicated a high level of intrinsic motivation. In the current study, this measurement showed good internal reliability, with a Cronbach’s alpha of 0.90. 

Flourishing. Flourishing was measured using the 14-item Mental Health Continuum-Short Form Scale (MHC-SF, [13,42]). An example item is “*How often did you feel that you belonged to a community*”. Items were rated on a six-point Likert scale. A high score indicated a high level of flourishing. In the current study, this measure showed good internal reliability, with a Cronbach’s alpha of 0.90. 

Social Capital. Social capital was measured using the Personal Social Capital Scale (PSCS, [43]). It consisted of three aspects of social capital, including structural social capital (e.g., “*In the past three months, how much did you participate in the activities of governmental, political, economic, and social group/organizations such as political parties, women’s groups, associations, volunteer groups, etc.*”), substantive social capital (e.g., “*In the past three months, how many of your friends and classmates had connections with others”*) and functional social capital (e.g., “*In the past three months, how much support did you receive from the help of your family members and relatives?”*). Items were rated on a five-point Likert scale. A higher score indicated a higher structural social capital. In the current study, this measure showed good internal reliability, with a Cronbach’s alpha of 0.89. 

### 2.3. Statistical Analysis

First, descriptive statistics and bivariate correlations were used to assess the relationship among all variables. SPSS Version 25.0 was used for data analysis, and statistical significance was set at *p* < 0.05. Next, the mediation analyses and moderated mediation analyses were performed using model 4 and model 7, respectively, in the PROCESS 3.2 macro. The significance of the mediation effect was also tested by bootstrapping the 95% confidence interval (95% CIs) of the indirect effect with 5000 repetitions. Specifically, a 95% confidence interval that does not include zero provides evidence for a significant indirect effect. 

## 3. Results

### 3.1. Common Method Bias

Harman’s single-factor test was used to evaluate the common method variance. If all items from each of the constructs load onto a single factor, or one general factor accounts for the majority of the covariance, this suggests that a substantial amount of common method variance is present [44]. In the present study, exploratory factor analysis (EFA) indicated that 17% (less than 40% of the critical value) of the total variation was extracted by one factor; thus, the treatment of common method biases is not present in this study.

### 3.2. Descriptive and Preliminary Analysis

Boys (N = 569, mean = 4.12, SD = 1.05) reported a higher level of flourishing than girls (N = 421, mean = 4.28, SD = 0.94), t = −2.72, *p* = 0.007. Age was negatively associated with intrinsic motivation, r = −0.14, *p* < 0.001. In addition, family income was negatively associated with social capital, r = −0.10, *p* = 0.002. Thus, sex, age and family income were included as covariates in further analyses. The inclusion of covariates, however, did not affect the significance of other paths in the model. The means, standard deviations and correlations of our study variables are shown in Table 1. 

Bivariate Pearson correlational analyses showed that all variables are strongly significantly associated. Regarding our first hypothesis, ego identity exploration was positively correlated with flourishing (r = 0.20, *p* < 0.001). As preliminary support for our proposed mediation model, ego identity exploration was positively correlated with intrinsic motivation, r = 0.20, *p* < 0.001, and intrinsic motivation was positively correlated with flourishing, r = 0.43, *p* < 0.001. 

### 3.3. Mediation Analyses

We first examined the indirect effect of intrinsic motivation on the associations between ego identity exploration and flourishing. Consistent with our hypotheses, ego identity exploration was associated with a higher level of intrinsic motivation, controlling for covariates, *b* = 0.29, *SE* = 0.04, *p* < 0.001, β = 0.22 (see Table 2). Further, intrinsic motivation was positively associated with flourishing, *b* = 0.49, *SE* = 0.04, *p* < 0.001, β = 0.39. In addition, ego identity exploration was also positively associated with flourishing, *b* = 0.21, *SE* = 0.05, *p* < 0.001, β = 0.13. The 95% bootstrapped CI of the indirect effect did not include zero, 95% CI [0.09, 0.19], suggesting this is a significant indirect effect. This model explained 7% of the variance in intrinsic motivation (a small effect) and a medium effect, that is, 20% in flourishing [45,46]. 

### 3.4. Moderated Mediation Analyses

Next, we tested the moderated mediation model which posits that social capital moderates the indirect effect of ego identity exploration on flourishing via intrinsic motivation (model type 7 for PROCESS). In this model, social capital was specified as a moderator variable conditioning the path from ego identification to intrinsic motivation (see Figure 1). As shown in Table 3, social capital moderated the effect of ego identity exploration on intrinsic motivation, *b* = −0.15, *SE* = 0.06, *p* = 0.01. Next, our results were also confirmed by the significant index of the moderated mediation test, *SE* = 0.03, 95% CI [−0.14, −0.01], which suggested that the influence of ego identity exploration on flourishing through intrinsic motivation is conditioned by social capital. This moderated mediation model explained 12% of the variance in intrinsic motivation (a medium effect) and 20% in flourishing (a medium effect). 

**Figure 2:**
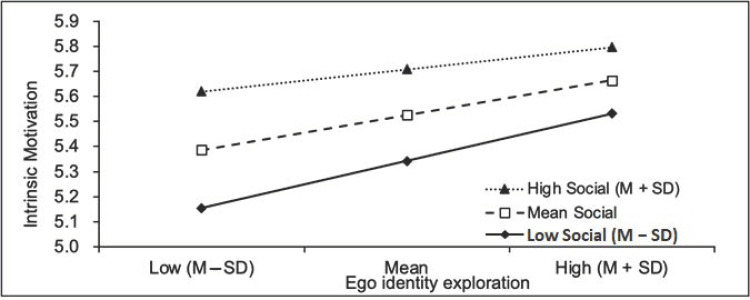
Simple slope analyses of the moderating effect of social capital.

As shown in Figure 2, the nature of the moderation was further explored using a simple slope analysis and conditioned as low (−1 SD), average (SD) and high (+1 SD). This analysis proved the effect significant for all groups. Specifically, compared with the individuals who had a higher level of social capital, individuals with a lower level of social capital tended to show a stronger positive relationship between ego identity exploration and intrinsic motivation.

## 4. Discussion

Flourishing is a necessary component of adolescents’ development, and exploring protective factors for adolescents’ flourishing is crucial. The findings from this study confirm the first hypothesis of the study (H1) by indicating that ego identity exploration played a positive role in promoting rural adolescents’ flourishing. For adolescents, exploring identities is a developmental task which may directly influence adolescents’ functioning [47]. During this process, they search for new interests, attitudes or values, which are likely to continuously reflect on the identities formed in childhood. This process may help adolescents to investigate their life in depth, which may enhance their autonomy and maintain their flourishing state. For instance, Waterman [48] and Luyckx et al. [49] claimed that ego identity exploration would help adolescents establish an identity commitment which was consistent with their personal characteristics and strengths. In line with this, Schwarz et al. [35] discovered that people who undertook in-depth exploration showed a high level of well-being. Thus, this exploration process does not support that the individual will only suffer a negative mental state and dysfunction. Instead, adolescents, in the period of searching, may also experience in-depth self-reflection which may contribute to their flourishing, as verified by the results of this study.

Our second hypothesis was also confirmed as there was an indirect effect of ego identity exploration on flourishing via intrinsic motivation (H2). Developing one’s identity is about answering the question, “*Who am I?*”, or synthesizing a confirmed viewpoint about *self*. In order to answer this question, the individual starts by finding out what attracts them, what they are skilled in and what roles they play in relationships, such as friendships [19,50]. The essence of ego identity exploration indicates vigorously searching for and assessing manifold values, interests, personal goals and relational roles [51], which may promote self-realization that conforms with the nature of intrinsic motivation [32,52]. Intrinsic motivation refers to an individual spontaneously engaging in activities for enjoyment and self-actualization, which, in turn, promotes their flourishing. Taken together, the individual explores and evaluates dimensional alternatives based on personal potential and dispositions, during which adolescents gradually enhance their autonomy by participating in activities which fulfill one’s interests and goals. As such, adolescents’ ego identity exploration process is positively related to their state of intrinsic motivation, which, in turn, contributes to their flourishing. 

Furthermore, this study also confirmed the third hypothesis (H3), that is, social capital plays a role in this indirect effect by showing a negative moderating effect on the relationship between ego identity exploration and adolescents’ intrinsic motivation. According to self-determination theory, the social context may be an influential factor for adolescents’ intrinsic motivation [53]. Interestingly, an overwhelming amount of social capital may weaken adolescents’ self-determination from ego exploration to motivation. For example, if parents exhibit excessive psychological control, children are forced to follow the parents’ rules, which impedes the growth of one’s intrinsic motivation. Large empirical studies have also illustrated the negative association between parental over-control and adolescents’ intrinsic motivation towards study [54,55]. On the contrary, parental support of autonomy is conducive to enhancing adolescents’ intrinsic motivation through allowing adolescents to make their own choices, express how they feel and explain why they want to engage in activities [56]. In rural China, adolescents whose social networks include their parents, teachers and even peers are influenced by traditional Chinese culture [57]. Thus, the expectations and social norms generated therein exert an influence on rural adolescents’ thoughts and behaviors; for instance, in order to obey the social norms or fulfill others’ expectations, rural adolescents may constrain themselves. From this perspective, a relatively low to moderate level of social capital could help rural adolescents to develop resilience as they explore their identities, thereby activating more growth in their intrinsic motivation. 

Furthermore, this study also found that age was negatively associated with rural adolescents’ intrinsic motivation, and females showed a higher level of flourishing than males. In terms of age differences in intrinsic motivation, they corresponded to the existing empirical studies [41,58]. These decreases are probably due to the cumulative impositions of constraints. For instance, along with the progress in growth, schools appear to have strengthened their control [59]. Meanwhile, the contents of the curriculum are becoming disconnected from daily life, thus reducing adolescents’ interest [60]. Next, there were no consistent results of gender differences in flourishing. Our findings indicate that females are in a higher state of flourishing. This can probably be explained by traditionally defined gender roles, in which females are expected to express emotional support and intimacy [61]. Our findings should be tested in future studies with samples from different cultures.

The results from this study have implications and contributions from both theoretical and practical perspectives. Regarding theoretical contributions, first, this study shifts the focus on rural adolescents’ development from a deficit-based approach to a strength-based approach by exploring their flourishing, which broadens and complements the existing empirical studies [7]. This study extends our understanding of the relationship among ego identity exploration, social capital and intrinsic motivation from personal and societal perspectives. 

In terms of practical contributions, the results of this study shed light on the importance of individual characteristics and the social context for rural adolescents’ development of flourishing. For example, parents and teachers could reinforce the communication with their children by decreasing their control over adolescents’ development and providing more support to their autonomy [62]. Considering the influence of traditional culture in rural China (e.g., Confucianism), rural adolescents sometimes do not pay much attention to their own personalities and desires but rather value fulfilling others’ expectations, such as those of their parents. As such, a need to promote adolescents’ flourishing by using strength-based intervention may be applied to rural Chinese adolescents (e.g., dig into rural adolescents’ characteristics, uniqueness, strengths and potentials, and then provide moderate support to adolescents to enhance their self-cognition). 

Several limitations of this study should also be noted. First, this study consists of cross-sectional survey research, and the results generated do not directly conclude causality relationships. Therefore, longitudinal research or experimental research could be conducted in the future to establish a causality result. Second, this study collected data from different schools and regions, but in the same province (Liaoning Province). Herein, the data obtained in this study represent only a specific area, which means we should be cautious when generalizing these findings. Future studies could acquire resources from other provinces to collect more multifarious data. Third, the measurement for ego identity exploration adopted in this study only examined ego identity exploration status and did not explore the different degrees of ego identity exploration status. Therefore, future studies could apply a more detailed measurement to examine ego identity exploration status. 

## 5. Conclusions

This study examined rural adolescents’ flourishing and explored several antecedent factors that influence their flourishing. Specifically, this study found that ego identity exploration contributed to rural adolescents’ flourishing, and intrinsic motivation could be a mediator within the relationship between ego identity exploration and flourishing. Additionally, this study found that social capital could display a moderating effect between ego identity exploration and intrinsic motivation. This study has several theoretical and practical contributions. First, this study shifts the traditional focus on rural adolescents to a positive viewpoint by highlighting the underlying mechanism of their flourishing. Second, this study integrates individual and social perspectives to search for potential protective factors for improving rural adolescents’ flourishing, thus depicting a more comprehensive picture of rural adolescents’ development. Finally, the findings of this study provide some practical suggestions to parents and school teachers regarding policy. 

## Figures and Tables

**Table 1:** Descriptive statistics and bivariate correlations in variables (N = 995).

	Mean	SD	1	2	3	4		
1. Sex	-	-	-					
2. Age	12.56	1.38	−0.06 *	-				
3. Family income	-	-	0.02	0.06	-			
4. Ego identity exploration	2.87	0.63	−0.12 ***	0.08 **	−0.02	-		
5. Intrinsic motivation	3.50	0.82	0.04	−0.14 ***	−0.02	0.20 ***	-	
6. Flourishing	4.18	1.01	0.09 **	−0.06	−0.04	0.20 ***	0.43 ***	-
7. Social capital	2.70	0.57	−0.07 *	−0.01	−0.10 **	0.29 ***	0.25 ***	0.32 ***

Note. CNY 1 = USD 0.16. * *p* < 0.05, ** *p* < 0.01, *** *p* < 0.001.

**Table 2:** Associations between ego identity exploration, intrinsic motivation and flourishing (N = 995).

	b	95% CI	t	SE	*p*	β
Paths						
Ego → Intrinsic	0.29	[0.21, 0.37]	7.15	0.04	<0.001	0.22
Intrinsic → Flourishing	0.49	[0.41, 0.56]	13.40	0.04	<0.001	0.39
Ego → Flourishing	0.21	[0.11, 0.30]	4.34	0.05	<0.001	0.13
Covariates						
Sex → Intrinsic	0.10	[−0.003, 0.20]	1.90	0.05	0.06	0.06
Age → Intrinsic	−0.09	[−0.13, −0.06]	−5.00	0.02	<0.001	−0.15
Income → Intrinsic	−0.01	[−0.11, 0.09]	−0.19	0.05	0.90	−0.01
Sex → Flourishing	0.17	[0.06, 0.29]	2.96	0.06	0.003	0.09
Age → Flourishing	−0.003	[−0.04, 0.04]	−0.14	0.02	0.89	−0.004
Income → Flourishing	−0.06	[−0.18, 0.05]	−1.07	0.06	0.29	−0.03
Total effects						
	0.35	[0.25, 0.44]	6.91	0.05	<0.001	
Indirect effects						
	0.14	[0.09, 0.19]	-	0.03	-	

Note. Ego = ego identity exploration. Intrinsic = intrinsic motivation. Income = family income. Sex (0 = males, 1 = females).

**Table 3:** Associations between ego identity exploration, intrinsic motivation, social capital and flourishing (N = 995).

	b	95% CI	t	SE	*p*
Paths of indirect effect					
Ego → Intrinsic	0.22	[0.14, 0.30]	5.28	0.04	<0.001
Intrinsic → Flourishing	0.49	[0.41, 0.56]	13.40	0.04	<0.001
Ego → Flourishing	0.21	[0.11, 0.30]	4.35	0.05	<0.001
Paths of moderation					
Social → Intrinsic	0.32	[0.23, 0.41]	7.06	0.05	<0.001
Ego × Social → Intrinsic	−0.15	[−0.26, −0.03]	−2.46	0.06	0.01
Covariates					
Sex → Intrinsic	0.11	[0.01, 0.20]	2.13	0.05	0.03
Age → Intrinsic	−0.09	[−0.12, −0.05]	−4.95	0.02	<0.001
Income → Intrinsic	0.02	[−0.07, 0,12]	0.50	0.05	0.62
Sex → Flourishing	0.17	[0.06, 0.29]	2.96	0.06	0.003
Age → Flourishing	−0.003	[−0.04, 0.04]	−0.14	0.02	0.89
Income → Flourishing	−0.06	[−0.18, 0.05]	−1.07	0.06	0.29
Conditional direct effect					
M − SD	0.30	[0.19, 0.41]	5.46	0.05	<0.001
M	0.22	[0.14, 0.30]	5.28	0.04	<0.001
M + SD	0.13	[0.03, 0.24]	2.63	0.05	0.01
Conditional indirect effects					
M − SD	0.15	[0.08, 0.21]	-	0.03	-
M	0.11	[0.06, 0.15]	-	0.02	-
M + SD	0.07	[0.01, 0.12]	-	0.03	-

## Data Availability

The data used in this research are available on request from the corresponding author. The data are not publicly available due to restrictions.
